# Higher 1-year risk of implant removal for culture-positive than for culture-negative DAIR patients following 359 primary hip or knee arthroplasties

**DOI:** 10.5194/jbji-7-143-2022

**Published:** 2022-07-06

**Authors:** Joyce van Eck, Wai-Yan Liu, Jon H. M. Goosen, Wim H. C. Rijnen, Babette C. van der Zwaard, Petra Heesterbeek, Walter van der Weegen, the further members of Regional Prosthetic Joint Infection Group

**Affiliations:** 1 Department of Orthopedic Surgery, Radboud University Medical Center, Nijmegen, the Netherlands; 2 Department of Orthopedic Surgery & Trauma, Catharina Hospital, Eindhoven, the Netherlands; 3 Department of Orthopedic Surgery & Trauma, Máxima MC, Eindhoven, the Netherlands; 4 Department of Orthopedic Surgery, Sint Maartenskliniek, Nijmegen, the Netherlands; 5 Department of Orthopedic Surgery, Jeroen Bosch Hospital, `s-Hertogenbosch, the Netherlands; 6 Sports & Orthopedics Research Centre, St. Anna Hospital, Geldrop, the Netherlands; ➕ A full list of authors appears at the end of the paper

## Abstract

**Background and purpose**: To date, the value of culture results after debridement, antibiotics, and implant retention (DAIR) for early (suspected) prosthetic joint infection (PJI) as risk indicators in terms of prosthesis retention is not clear. At the 1-year follow-up, the relative risk of prosthesis removal was determined for culture-positive and culture-negative DAIR patients after primary total hip or knee arthroplasty. The secondary aim of this work was to explore differences in patient characteristics, infection characteristics, and outcomes between these two groups. **Methods**: A retrospective regional registry study was performed in a group of 359 patients (positive cultures: n=299 ; negative cultures: n=60 ) undergoing DAIR for high suspicion of early PJI in the period from 2014 to 2019. Differences in patient characteristics, the number of deceased patients, and the number of subsequent DAIR treatments between the culture-positive and culture-negative DAIR groups were analysed using independent t tests, Mann–Whitney U tests, Pearson's chi-square tests, and Fisher's exact tests. **Results**: The overall implant survival rate following DAIR was 89 %. The relative risk of prosthesis removal was 7.4 times higher (95 % confidence interval (CI) 1.0–53.1) in the culture-positive DAIR group (37 of 299, 12.4 %) compared with the culture-negative DAIR group (1 of 60, 1.7 %). The culture-positive group had a higher body mass index ( p=0.034 ), a rate of wound leakage of >10 d ( p=0.016 ), and more subsequent DAIR treatments ( p=0.006 ). **Interpretation**: As implant survival results after DAIR are favourable, the threshold to perform a DAIR procedure for early (suspected) PJI should be low in order to retain the prosthesis. A DAIR procedure in the case of negative cultures does not seem to have unfavourable results in terms of prosthesis retention.

## 1 Introduction

Prosthetic joint infection (PJI) is a devastating complication, with an incidence of 0.5 %–2 % after primary knee arthroplasty and 0.5 %–1.0 % after primary hip arthroplasty (Edwards et al., 2009; Namba et al., 2013). In the case of a suspected early PJI, DAIR (debridement, antibiotics, and implant retention) treatment is recommended (Trampuz and Zimmerli, 2005​​​​​​​; Mühlhofer et al., 2017; Sousa and Abreu, 2018​​​​​​​; Barros et al., 2019). After initial intravenous (IV) antibiotic treatment, oral anti-biofilm combination therapy is administered for up to 3 months, based on intraoperative culture results (Osmon et al., 2013). Previous studies evaluating DAIR procedures have shown a prosthesis retention rate of 57 %–89 %, where the success of prosthesis retention is influenced by comorbidity, symptomatology, type of microorganism, and timing of the DAIR procedure in relation to the index surgery (Kuiper et al., 2013; Jacobs et al., 2019).

From the literature, it is not clear if there is a difference in outcome for patients with positive cultures after a DAIR procedure compared with patients with negative cultures. A meta-analysis of culture-positive and culture-negative infection procedures that included a heterogeneous group (both DAIR and revision procedures) of 283 patients did not show a clear difference in reinfection and cumulative survival rate between the groups (Reisener and Perka, 2018​​​​​​​).

The purpose of this study was to determine if there is a difference in prosthesis retention between culture-positive and culture-negative DAIR patients using a local registry on DAIR procedures after primary hip and knee arthroplasty. The secondary aim of this work was to explore differences in patient characteristics, infection characteristics, and outcomes (number of DAIR procedures performed and number of patients deceased) between these two groups. The hypothesis is that the culture-negative DAIR group will show better results with respect to implant retention at the 1-year follow-up. This is based on the assumption that a patient with a non-infected (culture-negative) prosthesis has a higher probability of prosthesis retention.

## 2 Methods

### 2.1 Study design, setting, and patient selection

**Figure 1:**
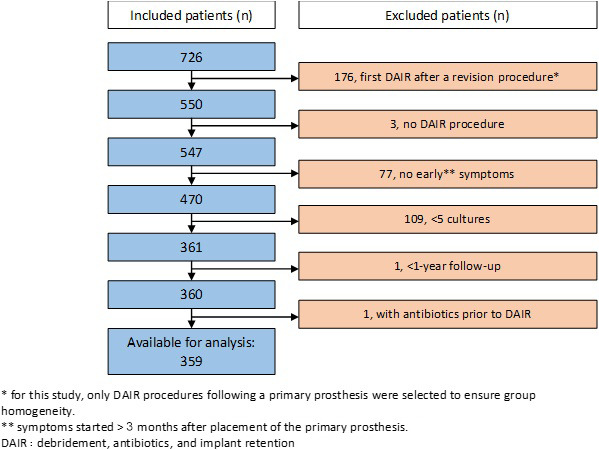
Flow diagram of the DAIR-treated patients included in the study.

A retrospective registry study was performed. A regional collaboration of eight hospitals, covering a region in the south-eastern Netherlands, committed to a standardized diagnostic and treatment protocol for suspected PJI. All suspected PJI cases were recorded in a regional database (Kamp et al., 2019). The regional hospitals included one academic hospital, five regional hospitals, one specialized hospital, and one private clinic. A case was considered as a suspected PJI using our standardized protocol and Wagenaar et al. (2019) based on at least one of the following items: clinical signs of infection (temperature >38.0 ${}^{\circ }$ C, pain, swelling, and redness of the wound), persistently elevated laboratory infection rates (C-reactive protein – CRP, erythrocyte sedimentation rate – ESR, and/or leukocytes), and persistent wound leakage (longer then 7–10 d). PJI cases were classified into early ( <3 months after implantation), delayed/low-grade (3–24 months after implantation), and late ( >24 months after implantation) infection. All patients who underwent a DAIR procedure after a total primary hip or knee prosthesis during the period from 2014 to 2019 were registered. Patients with a delayed/low-grade or late infection, non-acute symptoms, less than five intraoperative cultures during the index DAIR, and/or patients who had already received antibiotics prior to the index DAIR were excluded (Fig. 1). Our decision to exclude non-early infections ( >3 months) was based on Schafroth et al. (2003). DAIR procedures were considered culture-positive if two or more deep-tissue biopsies showed a positive culture for the same microorganism. All culture-positive DAIR procedures were allocated to the “positive DAIR” group, and all culture-negative DAIR procedures were assigned to the “negative DAIR” group. All of the review committees of participating hospitals granted approval, and the study was carried out in accordance with the applicable legislation, including review by an accredited research ethics committee (2020–7193).

### 2.2 PJI treatment and variables

DAIR was repeated if clinical symptoms and laboratory signs did not improve within 10 d of the first DAIR procedure, according to the regional protocol, which allowed a maximum of two DAIR procedures. If the infection was still present after these DAIR procedures, the decision and arrangements to remove the implant were made by the treating surgical team (Kamp et al., 2019). Data regarding patient characteristics, type of surgery (primary hip or knee prosthesis), time between the index surgery and the DAIR procedure, features of infection (persistent wound leakage or fever), infection parameters (CRP and ESR), exchange of components during the DAIR procedure, and microbiology cultures were collected from the regional database.

The primary outcome of this study was the retention of the fixed parts of the prosthesis 1 year after (the first) DAIR procedure in patients with a primary total knee arthroplasty or total hip arthroplasty. A successful outcome was defined as retention of the fixed parts of the prosthesis 1 year after the DAIR procedure. A case was considered to be a “retained prosthesis” if all clinical (including CRP) and radiographic signs of infection were absent at the 1-year follow-up. Patients who were deceased at the follow-up with the prosthesis still in situ were not considered to be a “failure”. Secondary outcomes were patient characteristics (gender age; body mass index, BMI; American Society of Anesthesiology, ASA, score; smoking; and presence of diabetes mellitus or rheumatoid arthritis), infection characteristics (persistent wound leakage and body temperature >38.0 ${}^{\circ }$ C), number of DAIR procedures performed for the same implant, and number of patients deceased within 12 months.

### 2.3 Statistical analysis

Kolmogorov–Smirnov tests were used to assess the normality of the data. The mean and standard deviation (SD) were used to present normally distributed data, and the median and interquartile range (IQR) were used for non-parametric data. Differences between the positive and negative DAIR groups were analysed using independent t tests or Mann–Whitney U tests for continuous variables and Pearson's chi-square​​​​​​​ tests or Fisher's exact tests for categorical data. All analyses were performed using IBM SPSS Statistics (version 25.0, IMB Corp, Armonk, NY, USA). A p value of less than 0.05 was considered to be statistically significant.

## 3 Results

At the eight participating hospitals, DAIR procedures were performed on 750 patients after a hip or knee prosthesis between January 2014 and December 2019 (Fig. 1); of these 750 patients, 359 (228 primary total hip prostheses and 131 primary total knee prostheses) were included in our analyses. The cultures obtained during the DAIR procedures were positive in 299 cases and negative in 60 cases.

**Table 1:** Patient characteristics of DAIR-treated patients ( n=359 ).

	Positive DAIR group	Negative DAIR group	p value
	n=299	n=60	
Male sex, n (%) ${}^{a}$	178 (59.5)	37 (61.7)	0.758
Age in years, mean (SD) ${}^{b}$	67.7 (11.1)	67.9 (9.9)	0.925
BMI, median (IQR) ${}^{c}$	30.2 (5.6)	28.5 (5.2)	0.034
ASA score ${}^{d}$			0.640
Score 1, n (%)	37 (12.4)	9 (15.0)	
Score 2, n (%)	203 (67.9)	42 (70.0)	
Score 3, n (%)	59 (19.7)	9 (15.0)	
Diabetes mellitus, n (%) ${}^{d}$	22 (7.4)	4 (6.7)	1.000
Smoking, n (%) ${}^{d}$	36 (12.0)	7 (11.7)	0.935
Rheumatoid arthritis, n (%) ${}^{d}$	29 (9.7)	11 (18.3)	0.052
Type of index surgery ${}^{a}$			0.395
Primary total knee replacement, n (%)	112 (37.5)	19 (31.7)	
Primary total hip replacement, n (%)	187 (62.5)	41 (68.3)	
Type of fixation ${}^{a}$			0.505
Uncemented, n (%)	83 (27.8)	21 (35.0)	
Cemented, n (%)	196 (65.6)	36 (60.0)	
Hybrid, n (%)	20 (6.7)	3 (5.0)	
Urgency of index surgery ${}^{d}$			0.361
Elective, n (%)	291 (97.3)	60 (100.0)	
Acute, n (%)	8 (2.7)	0 (0.0)	
Previous surgery on the same joint, n (%) ${}^{a}$	30 (10.0)	6 (10.0)	0.994
Post-operative complication of index surgery, n (%) ${}^{a}$	32 (10.7)	5 (8.3)	0.582
Suspected infection characteristics			
Persistent wound leakage, n (%) ${}^{a}$	212 (70.9)	33 (55.0)	0.016
Temperature >38.0 ${}^{\circ }$ C, n (%) ${}^{a}$	52 (17.4)	13 (21.7)	0.433
CRP before DAIR treatment, median (IQR) ${}^{c}$	100.42 (114.0)	72.3 (85.2)	0.108
ESR before DAIR treatment, median (IQR) ${}^{c}$	45.2 (27.9)	47.7 (33.3)	0.957
Days between index surgery and DAIR treatment, median (IQR) ${}^{c}$	22.8 (13.8)	29.5 (60.6)	0.103

The abbreviations used in the table are as follows: DAIR – debridement, antibiotics, and implant retention; BMI – body mass index; CRP – C-reactive protein; ESR – erythrocyte sedimentation rate. ${}^{a}$ Chi-square test. ${}^{b}$ t test. ${}^{c}$ Mann–Whitney U test. ${}^{d}$ Fisher exact test.

**Table 2:** Outcome measures of DAIR-treated patients ( n=359 ).

	Positive DAIR group	Negative DAIR group	p value
	n=299	n=60	
Exchange of components during DAIR, n (%) ${}^{a}$	204 (68.2)	38 (63.3)	0.442
Second DAIR, n (%) ${}^{b}$	105 (35.1)	10 (16.7)	0.006
Prosthesis retention after 1 year, n (%) ${}^{a}$	262 (87.6)	59 (98.3)	0.004
Deceased within 1 year of first DAIR, n (%) ${}^{b}$	11 (3.7)	0 (0.0)	0.223

DAIR: debridement, antibiotics, and implant retention. ${}^{a}$ Chi-square test. ${}^{b}$ Fisher exact test.

Overall, prosthesis retention 1 year after DAIR was 89.4 % (321 of 359 patients). In the positive DAIR group, 37 (12.4 %) patients had their prosthesis removed within 1 year of the DAIR procedure versus 1 patient (1.7 %) in the negative DAIR group (relative risk, RR, of 7.4, 95 % confidence interval (CI) 1.039–53.072) (Table 2). Excluding patients who had their prosthesis removed for aseptic reasons ( n=2 ) did not influence the relative risk (RR of 7.3, 95 % CI 1.025–52.347).

Patients in the positive DAIR group had a 2.7 point higher body mass index (BMI) than patients in the negative DAIR group ( p=0.034 ). Furthermore, a higher percentage of patients with prolonged wound leakage of more than 10 d was observed in the positive DAIR group ( p=0.016 ) (Table 1). The number of second DAIR procedures performed was significantly higher in the positive DAIR group ( n=105,35.1 %) than in the negative DAIR group ( n=10 , 16.7 %; p=0.006 ). In the positive DAIR group, 44 (41.9 %) of the second DAIR procedures also had two or more positive cultures. In 10 patients, a second DAIR was performed after a culture-negative first DAIR. In five of these cases, two or more positive cultures were found in the second DAIR procedure. A total of 11 patients (3.7 %) in the positive DAIR group were deceased at the 1-year follow-up, whereas none of the negative DAIR group were deceased. An overview of the patient characteristics and outcome measures analysed for the positive and negative DAIR groups are summarized in Tables 1 and 2. No data were missing.

The patient with negative cultures obtained during the DAIR who ultimately had their total knee prosthesis removed needed a second DAIR for persistent wound leakage. Cultures obtained from this second DAIR were positive for *Staphylococcus aureus* and *Staphylococcus epidermis*. Due to persistent signs of infection, this ultimately led to a two-stage revision. In two patients, the acetabular component was revised for recurrent dislocation. In both patients, clinical signs of infection were absent.

## 4 Discussion

In this retrospective registry study, patients with two or more positive cultures after the index DAIR had a 7.4 (CI 1.0–53.1) higher risk of prosthesis removal 1 year after the procedure compared with patients with negative cultures. The overall success rate based on prosthesis retention was 89.4 % at the 1-year follow-up. This is comparable to other studies reporting a success rate ranging from 41 % to 95 % (Kazimoglu et al., 2015; Bergkvist et al., 2016; Romano et al., 2014; Reisener et al., 2018; Jacobs et al., 2019). A systematic review by Romano et al. (2016) reported a success rate of 44.9 %–52.0 % in a total of 796 DAIR patients based on prosthesis retention at an average of 4 years post-procedure. The success rate depended on the time frame between the surgical intervention and the start of symptoms. Jacobs et al. (2019) assessed prosthesis retention 1 year after DAIR in 20 culture-negative DAIR and 71 culture-positive DAIR patients, of which 85 % had their prosthesis retained. All of the 20 culture-negative DAIR patients had a successful outcome (defined as the absence of clinical and/or laboratory signs of infection and no removal of the prosthesis) at the 1-year follow-up. Jacobs et al. (2019) concluded that DAIR is the appropriate treatment for the suspicion of an early PJI, as culture-negative DAIR procedures were not related to complications during follow-up and the over-treatment of a suspected PJI apparently does not lead to implant failure. A systematic review by Reisener et al. (2018) included eight studies in which the negative-culture group had the same or an even better infection-free survival rate than the culture-positive group (Reisener et al., 2018). Both the study of Jacobs et al. (2019) and our results show that patients with positive cultures after a DAIR procedure have an increased risk of prosthesis removal within 1 year. Unfortunately, neither study has sufficient power to perform a multivariate analysis to determine the factors associated with prosthesis retention. When we excluded patients who had their prosthesis removed within 1 year of the procedure for aseptic reasons ( n=2 ), this only resulted in small changes in the relative risk and confidence intervals.

The second purpose of this research was to explore the differences in patient characteristics between the negative and positive DAIR groups. The positive DAIR group was associated with a higher BMI (30.2, compared with 28.5 in the negative DAIR group), a higher occurrence of persistent wound leakage, and more subsequent DAIR procedures compared with the negative DAIR group (Tables 1, 2). Similar findings have been observed in other studies. A 2- to 6-fold increased risk of PJI in patients with a BMI ≥35 kg m ${}^{-2}$ has been reported (Alvi et al., 2015; Lubbeke et al., 2016). Kremers et al. (2019) observed more wound leakage after total knee arthroplasty (TKA) or total hip arthroplasty (THA) in the PJI group compared with the control group. In concordance with literature describing the association of BMI and wound leakage with an increased probability of PJI, our study results also show that these factors are more present in the positive DAIR group.

An interesting finding from our study was that 5 out of 10 patients in the negative DAIR group who underwent a second DAIR had positive cultures. Four out of five of these patients had their implant retained at the 1-year follow-up. There is a possibility that a negative culture could be the result of suboptimal diagnostic properties of cultures and were never infected in the first place (Matsen Ko and Parvizi, 2016​​​​​​​). On the other hand, a (culture-negative) DAIR procedure could have introduced a new infection rather than treating one. In the case of doubt, before deciding to perform a DAIR, additional PJI diagnostics could be considered when, for example, the patient shows no extensive systemic signs of an infection, (Parvizi et al., 2013, McNally et al., 2021).

The main limitation of this study was a relatively low number of failures in the negative DAIR group which resulted in insufficient power to perform a multivariate analysis. This made further statistical exploration of contributing factors impossible. Nevertheless, the results show a clear pattern, and the findings of our study are in concordance with others. Furthermore, differences with respect to data interpretation at the participating centres could introduce variation in database entries. By using a standardized diagnostic and treatment protocol and by organizing regular meetings for data verification, we tried to minimize missing data or incorrect data entries. In addition, the multicentre design of this study ensures the generalizability of our results. Another limitation was that there were 109 patients who had less than five cultures and, therefore, did not meet the inclusion criteria. Earlier research by Kamp et al. (2019) showed that this was more often the case in the early years of the collaboration. Due to the retrospective nature of this study and the lack of studies with the same purpose, it is conceivable that unknown confounding factors exist. Therefore, we recommend that further research be conducted that considers possible influential factors such as comorbidity, symptomatology, type of microorganism, and timing of the DAIR procedure in relation to the index surgery (Kuiper et al., 2013; Jacobs et al., 2019).

In conclusion, overall, we observed 89.4 % prosthesis retention at the 1-year follow-up. Patients with a culture-positive first DAIR had a 7 times higher risk of prosthesis removal at the 1-year follow-up than patients with culture-negative first DAIR procedures. As implant survival results after DAIR are favourable, the threshold to perform a DAIR procedure for (suspected) early PJI should be low in order to retain the prosthesis. This can result in a culture-negative DAIR with a low complication rate.

## Data Availability

All data generated and analysed during this study are included in this published article (e.g. tables and figures)​​​​​​​ and are available from the corresponding author upon reasonable request.

## Team list

Further members of the Regional Prosthetic Joint Infection working group who participated in the registry are as follows: Stefan F. de Boer (VieCuri, the Netherlands), Dirk Das (St. Anna Hospital, the Netherlands), Johannes G. E. Hendriks (Máxima MC, the Netherlands), Robin W. T. M. van Kempen (Catharina Hospital, the Netherlands), Yvette Pronk (ViaSana, the Netherlands), Katja Saris (Radboudumc, the Netherlands), Tim Weernink (Bernhoven, the Netherlands).​​​​​​​
